# Relationship between frailty, nutrition, body composition, quality of life, and gender in institutionalized older people

**DOI:** 10.1007/s40520-022-02077-0

**Published:** 2022-02-11

**Authors:** S. K. Jyväkorpi, M. Lindström, M. H. Suominen, H. Kautiainen, K. Salminen, R. T. Niskanen, K. H. Pitkälä, H-M. Roitto

**Affiliations:** 1 University of Helsinki, Clinicum, Finland; 2 City of Helsinki Department of Social Services and Health Care, Geriatric Clinic, Helsinki Hospital, Helsinki, Finland

**Keywords:** Frailty, Institutionalized older people, Nutrition, Gender, Muscle mass, Health-related quality of life

## Abstract

Our aim was to explore the relationship between frailty, nutrition, body composition, and how gender modifies this relationship among long-term care facility residents. We further investigated how body composition correlates with health-related quality of life (HRQoL) in both genders. In all, 549 residents (> 65 years of age) were recruited from 17 long-term care facilities for this cross-sectional study. Demographic information, diagnoses, use of medications, and nutritional supplements were retrieved from medical records. Participants’ frailty status, cognition, nutritional status, HRQoL, and body composition were determined. Energy, protein, and fat intakes were retrieved from 1- to 2-day food diaries. The final sample consisted of 300 residents (77% women, mean age 83 years). The majority of participants, 62% of women and 63% of men, were identified as frail. Frail participants in both genders showed lower body mass index (*p* = 0.0013), muscle mass (MM) (*p* < 0.001), poorer nutritional status (*p* = 0.0012), cognition (*p* = 0.0021), and lower HRQoL (*p* < 0.001) than did prefrail participants. Women had higher fat mass, whereas men exhibited higher MM. The HRQoL correlated with the MM in both women, *r* = 0.48 [95% CI 0.38, 0.57] and men *r* = 0.49 [95% CI 0.38, 0.58]. Interventions aimed at strengthening and retaining MM of long-term residents may also support their HRQoL.

## Introduction

The age-related loss of physical performance and functioning often results from numerous clinical and subclinical conditions, such as frailty and sarcopenia [1, 2]. Frailty is a multifactorial geriatric syndrome characterized by decreased reserve and is a leading contributor to functional decline and early mortality in older adults [3]. The characteristics of the frailty phenotype include weight loss, muscle weakness, exhaustion, slow walking speed, and low physical activity [2]. Sarcopenia is closely associated with frailty and is characterized by low muscle strength, low muscle mass (MM), and poor muscle quality, as well as reduced physical performance [1]. Malnutrition, particularly inadequate energy and protein intake, is a key element associated with loss of MM and physical functioning in older people [4, 5]. All frailty, sarcopenia, and malnutrition increase the risk of falling, use of healthcare services, poor quality of life, institutionalization, and mortality, and are often interrelated [1–3, 6, 7]. Recently poor muscle function associated with sarcopenia was linked with late-life cognitive impairment [8], which is often encountered also in frail and malnourished older people [3, 6]. Indeed, malnutrition, frailty, and sarcopenia share a common pathophysiology and are manifested by weight loss, which is a typical characteristic in all these situations [1, 2, 6, 9]. In addition to weight loss, frailty and sarcopenia are also associated with other characteristics of poor nutrition, such as inadequate energy or protein intake and poor diet quality, whereas sufficient intake of nutrients may have an ancillary role in musculoskeletal health [1, 2, 6, 7, 9–12].

Institutionalized older people are often frail, sarcopenic, and malnourished [1, 13, 14, 16]. Two systematic reviews and meta-analyses showed that malnutrition and malnutrition risk among institutionalized older people varied from 14 to 21% and from 45 to 53%, respectively [13], and the prevalence of frailty between 19 and 76% [14]. In various studies, low health-related quality of life (HRQoL) has been inversely associated with sarcopenia, frailty, and malnutrition in long-term residents [15–17]. However, little is known about the association between frailty, body composition, nutrition, and HRQoL in long-term residents. Since our participants were a unique group of long-term care residents living their final years, we aimed at determining how frailty, body composition, nutritional status, nutrient intake, and HRQoL are related, and how gender modifies this relationship in older long-term care residents. We also aimed at determining how body composition is correlated with the HRQoL.

## Materials and methods

In all, 549 volunteer residents were recruited for this cross-sectional study from a sample of three nursing homes and 14 assisted living facilities in Helsinki. These facilities include group homes for older people with dementia. In all the institutions registered nurses were in charge of the wards and constant 24/7 assistance was available. Participants’ assessments took place between April 2017 and August 2018.

The inclusion criteria for the present study were as follows: age ≥ 65 years, living permanently in institutional care, sufficient information available on demographic factors, frailty status determined according to Fried’s phenotype criteria, records of a 1–2-day food diary, information on 15-D HRQoL [18], and body composition measurement with bioimpedance spectroscopy.

In each ward, trained nurses collected the data. The participants’ weights were measured. Their heights were obtained from the medical records, and body mass index (BMI) was calculated as weight divided by height squared (kg/m^2^). Information on the residents’ demographic information, diagnoses, and use of medications and nutritional supplements were retrieved from medical records. Frailty status was determined, using modified Fried phenotype frailty criteria [2] as follows: (1) Unintentional weight loss > 5% during the past 3 years (yes/no). (2) Exhaustion—nurse-reported or self-reported low energy levels most or all of the time during the previous 4 weeks. (3) Low physical activity was assessed by a question on whether the participant exercised regularly on a weekly basis (yes/no). (4) Slowness – based on the gate speed using a 4-m walk time from the Short Physical Performance Battery test (SPPB) [19] – was defined as < 0.85 m/s. (5) Physical weakness as a nurse-reported or self-reported difficulty (not at all = 0) of carrying or lifting a grocery bag or an object weighing about 5 kg. Those who did not fulfill any frailty criteria were defined as robust, those who filled 1 or 2 of the above criteria were classified as prefrail, and those who met ≥ 3 criteria were classified as frail. Since only two residents were defined as robust, they were classified into the prefrail group. The cognitive status of the residents was measured, using the Mini Mental State Examination (MMSE) [20]. Nutritional status was assessed, using the Mini Nutritional Assessment (MNA) long version [6]. The body composition of the participants was measured, using a bioimpedance spectroscope performed by a trained nurse or researchers (KS, RN,HMR) with a single-channel, tetra polar device (SFB7, ImpediMed Ltd., Eight Miles Plains, Queensland, Australia) that scans 256 frequencies between 4 and 1000 kHz. The measurements were performed according to standard measurement practices, in which the person was sitting or lying down and four electrodes were attached to the right hand and ankle. Values processed by the software were used to determine MM and fat mass of the participants. Muscle% and fat % were further calculated (MM/body weight × 100%, fat mass/body weight × 100%).

We used the 15-D instrument for measuring HRQoL [18]. The instrument has 15 dimensions, which include mobility, vision, hearing, breathing, sleeping, eating, speech, excretion/elimination, usual activities, mental function, discomfort and symptoms, depression, distress, vitality, and sexual activity. The 15-D can be completed during a conversation with the resident, but also by proxy who knows the resident well. A score of 0 indicates the poorest HRQoL and 1 indicates the best interviewed for the 15-D if the subject was unable to respond due to poor cognition.

Participants’ energy, protein, and fat intakes were determined from 1- to 2-day food diaries kept by the ward nurses. Prior to the data collection, the nurses participated in comprehensive training sessions on how to fill the food diaries for the residents held by study’s investigators (SKJ, MS). The food diaries were analyzed, using AivoDiet dietary software (version 2.2.0.0, Aivo Oy, Turku, Finland), which contains the Fineli Food Composition database Release 16 (2013), including foods and recipes for the typical Finnish mixed dishes that are customarily served in long-term care. The instruction was to record all the foods and beverages consumed by the resident. The nurses estimated portion sizes, using household measures. For prepacked products, the exact brand and product name were required.

Other nutrition-related questions included information on estimates of the amounts of foods consumed with a question “How much does the resident eat of the main meal on average?” with five response options: “eats only a little, eats less than half, eats half the meal, eats most of the meal, or eats all or nearly all of the meal.” The responses “eats only a little” and “eats less than half” were dichotomized to “eats less than half,” and the response “eats half the meal,” “eats most of the meal,” or “eats all or nearly all of the meal” as eats more than half. The portion sizes were compared model images of the food portions. We also asked whether the participants regularly ate snacks (yes/no).

The participants were divided into groups according to their frailty status (prefrail, frail) and gender. Background and nutritional variables were classified into these groups accordingly. Relationship between the gender and frailty status of background characteristics and body composition were evaluated using two-way analysis of variance and logistic models. In the case of violation of the assumptions (e.g., non-normality) for continuous variables, a bootstrap-type method or Monte Carlo p-values (small number of observations) for categorical variables were used. Relationships between muscle mass and Health-related quality of life was analyzed by using linear regression model. Correlation coefficients were calculated by the Pearson method. The normality of the variables was evaluated graphically and by the Shapiro–Wilk W test. The Stata 16.0 (Stata Corp LP; College Station, TX, USA) statistical program was used for the analysis.

## Results

In total, 300 residents (77% women), of whom we received all the information required, were eligible to participate in this study. Almost all (99%) of the participants in this study were at least prefrail. Frailty was identified in 62% of the participants (62% of women and 63% of men). Those identified as frail did not differ from prefrail participants in age, years of education, or frequency of the most common chronic diseases (Table 1). The BMI was higher in both genders in prefrail participants, while malnutrition and malnutrition risk were higher in frail individuals. Malnutrition was more prevalent in prefrail and frail females than males. The number of medications was lower in those identified as frail than in prefrail in both genders (women 8.8 vs. 8.3; men 10.2 vs. 8.0, *p* = 0.0011). HRQoL and cognition were higher, whereas the need for help was lower in the prefrail group than in the frail group in both genders.

**Table 1:** Baseline characteristics according to frailty status in women and men in institutionalized care

Frailty status Characteristics	Women		Men		*p*-value		
	Prefrail *n* = 89	Frail *n* = 143	Prefrail *n* = 25	Frail *n* = 43	Sex	Frail	Interaction
Age, mean (SD)	83 (8)	83 (8)	82 (8)	83 (7)	0.71	0.92	0.79
BMI, kg/m^2^ (SD)	27.4 (5.0)	25.5 (5.1)	28.1(4.0)	26.5 (4.2)	0.25	0.013	0.85
Education < 8 years, *n* (%)	38 (43)	61 (43)	12 (48)	15 (35)	0.84	0.35	0.34
Dementia, *n* (%)	74 (83)	115 (80)	19 (76)	36 (84)	0.76	0.67	0.35
Diabetes, *n* (%)	20 (22)	24 (17)	5 (20)	11 (26)	0.58	0.95	0.33
Stroke, *n* (%)	11 (12)	28 (20)	11 (44)	17 (40)	< 0.001	0.57	0.25
Lung disease, n (%)	6 (7)	18 (13)	2 (8)	8 (19)	0.50	0.088	0.77
Cancer, *n* (%)	12 (13)	18 (13)	3 (12)	4 (9)	0.60	0.69	0.82
Musculoskeletal system disease, *n* (%)	22 (25)	37 (26)	5 (20)	5 (12)	0.099	0.44	0.35
MNA, *n* (%)					0.010	0.012	0.23
Normal	20 (27)	14(12)	8 (33)	9 (26)			
Malnutrition risk	50 (67)	81 (68)	16 (67)	23 (68)			
Malnutrition	5 (7)	25 (21)	0 (0)	2 (6)			
Medications, mean (SD)	8.8 (3.7)	8.3 (3.6)	10.2 (3.1)	8.0 (3.7)	0.31	0.011	0.099
Health-related quality of life 15-D mean score (SD)	0.68 (0.11)	0.59 (0.12)	0.72 (0.10)	0.60 (0.11)	0.12	< 0.001	0.50
MMSE, score (SD)	14.9 (6.5)	12.3 (7.8)	15.4 (5.3)	13.0 (7.1)	0.59	0.021	0.92
Needs lots of help in daily chores, *n* (%)	34 (38)	102 (71)	14 (56)	33 (77)	0.10	< 0.001	0.47

*BMI* body mass index, *MNA *Mini Nutritional Assessment, *MMSE* Mini Mental State Examination, *SD* standard deviation

The energy, protein, and fat intakes did not differ between the frail and prefrail groups. However, we did observe a gender difference; frail women had lower energy and protein intakes than did the prefrail women, whereas frail men had higher intake of energy and protein (interaction *p* = 0.044 for energy, and *p* = 0.033 for protein) (Table 2). Men were more likely to consume higher amounts of foods than women (*p* < 0.001).

**Table 2:** Nutritional characteristics according to frailty status of women and men in institutionalized care

	Women		Men		*p*-value		
Frailty status Nutritional characteristics	Prefrail *N* = 89	Frail *N* = 143	Prefrail *N* = 25	Frail *N* = 43	Sex	Frail	Interaction
Energy, kcal (SD)	1667 (408)	1527 (378)	1768 (345)	1846 (338)	< 0.001	0.57	0.044
Total protein, g	58 (17)	51 (15)	62 (13)	64 (15)	< 0.001	0.36	0.033
g kg^−1^ BW d^−1^ (SD)	0.9 (0.3)	0.8 (0.3)	0.7 (0.1)	0.8 (0.2)	0.15	0.34	
Total fat, g (SD)	63 (21)	60 (18)	67(16)	75 (16)	< 0.001	0.36	0.061
SFA, g	30 (11)	30 (10)	32 (10)	36 (10)	0.003	0.15	0.093
MUFA, g	19 (7)	18 (6)	20 (5)	22 (5)	< 0.001	0.84	0.070
PUFA, g	7 (3)	6 (2)	8 (2)	8 (3)	< 0.001	0.37	0.13
Nutritional supplements, *n* (%)	17 (19)	31 (22)	3 (12)	7 (16)	0.27	0.53	0.81
Eats snacks, *n* (%)	75 (84)	105 (73)	23 (92)	38 (88)	0.060	0.25	0.79
Eats, *n* (%)					< 0.001	0.72	0.53
Little	18 (20)	35 (24)	1 (4)	4 (9)			
Normal	65 (73)	103 (72)	21 (84)	31 (72)			
A lot	6 (7)	5 (3)	3 (12)	8 (19)			

*BW *body weight, *SFA* saturated fatty acids, *MUFA* monounsaturated fatty acids, *PUFA* polyunsaturated fatty acids, *SD* standard deviation

**Fig. 1:**
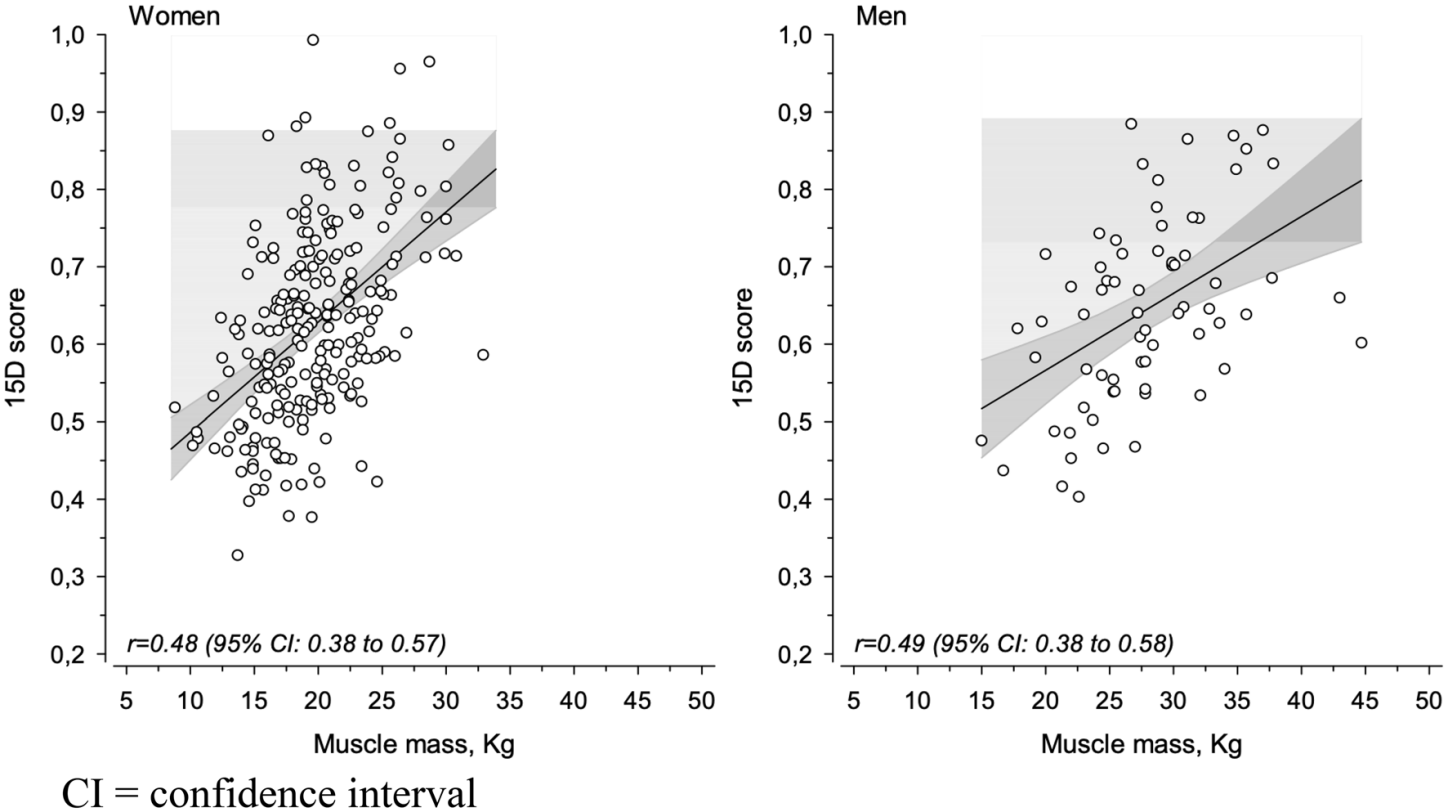
Correlation between muscle mass and health-related quality of life in women and men in institutionalized setting. *CI *confidence interval

Frailty was inversely associated with total MM in both genders (*p* < 0.001) (Table 3). Men also exhibited more MM in general than women (*p* < 0.001), whereas neither total fat mass nor fat% differed between frailty statuses. Fat% was higher and muscle % lower in women (*p* < 0.001) than in men. The HRQoL correlated with total MM in both genders, in women *r* = 0.48 [95% confidence interval (95% CI) 0.38, 0.57] and men *r* = 0.49 [95% CI 0.38, 0.58] (Fig. 1).

**Table 3:** Body composition according to frailty status in women and men in institutionalized care

Frailty status Body composition	Women		Men		Sex	Frailty status	Interaction
	Prefrail *n* = 89	Frail *n* = 144	Prefrail *n* = 25	Frail *n* = 43			
Total muscle mass, kg (SD)	21.3 (4.2)	18.6 (3.9)	30.2 (4.3)	26.4 (6.2)	< .001	< .001	0.38
Total fat mass, kg (SD)	29.5(11.1)	27.3(11.4)	28.6(9.3)	25.2(12.1)	0.079	0.35	0.73
Fat % (SD)	40.8(9.4)	41.7(10.9)	33.4(8.1)	32.9(12.4)	< 0.001	0.88	0.66
Muscle % (SD)	30.8(5.0)	30.2(6.1)	36.0(4.6)	36.2(7.7)	< 0.001	0.80	0.66

## Discussion

In our study, almost all of the residents were at least prefrail, while the majority was frail. Frailty was inversely associated with BMI, MM, nutritional status, and cognition. Frail residents also reported having lower HRQoL, and they were more dependent than the prefrail residents. MM was a strong predictor of HRQoL in both genders. Gender difference was most notable in nutrition-related issues; female residents were more likely to be malnourished or at risk of malnutrition, had lower energy and nutrient intakes, and less favorable body composition than the male residents.

Prefrailty and frailty were very commonly found in our study, since practically all of our participants were at least prefrail. In previous studies, frailty in nursing homes or in other long-term institutions has also been prevalent, but has varied considerably (1.7–72.5%), depending on the tool used to identify frailty [21]. Fried’s frailty phenotype definition that we used in our study is associated with several clinical indicators, suggesting high levels of disability and increased risk of developing major clinical consequences, which seem appropriate for this vulnerable population [16]. Other validated, well-known frailty tools include, for example, simple FRAIL questionnaire that also has five questions similar to Fried’s frailty tool, but it is even more simple [2, 22] and PRISMA 7 which includes seven questions [23]. Rockwood et al.’s Clinical Frailty Scale, which evaluates specific domains, including comorbidity, function, and cognition to generate a frailty score, is also often used to identify frailty [24]. We think that all these frailty tools would have given similar results in our population of older residents. A systematic review and meta-analysis by Kojima [14] of nine studies with a total of 1373 residents revealed that the prevalence of frailty, including prefrailty, ranged from 19% to 75.6%. The high prevalence of prefrailty and frailty in our study may have been due to the fact that during recent decades in Finland the public policy has been to reduce institutionalized care for older people [25]. Thus, only those who have very severe dementia, mobility disability, or other severe health complications due to multiple chronic diseases are offered a place in a nursing home or assisted living facility type of long-term care. As a result, the populations residing in these institutions are usually living the last years of their lives and cannot cope with living at home even with help of a caregiver or intensive home care which would explain the high prevalence of prefrailty and frailty in our study.

There was a gender difference between women and men in nutrition in our study. Women in our study were more likely to be malnourished than men. Moreover, women’s energy, protein, and fat intakes were lower than those of men, which is probably due to the higher energy consumption of men. However, since frail female residents showed lower energy and protein intakes than did prefrail female residents, contrasting situations were observed in men. Frail males received more energy and protein than did prefrail male residents. We speculated that frail males could receive more nutritional care than frail females or prefrail males, and be put on more energy––and protein––dense special diets. Very few published articles are available on the gender differences in healthcare. In a previous study, male residents with Alzheimer’s disease (AD) were more likely to receive intervention programs for mood, behavior, and cognitive loss than female residents with AD [26]. Frail males could thus receive more nutritional care interventions than frail females. However, the number of men in this study was relatively low (23% of all participants) which could have affected the interpretation of the results observed. In any case, gender aspect in long-term care is an interesting subject with very few published studies available, and we strongly encourage other researchers to explore this important subject in different populations and longitudinal studies.

Men are well known for having about one-third more MM than women, and their muscle loss with aging occurs at a slower pace than in women [27]. In our study, MM showed strong correlation with HRQoL among long-term residents in both genders. MM was a better predictor of 1-year mortality than BMI [28]. In a study by van Ancum et al. [29], lower MM was associated with posthospitalization falls. Similarly, lower skeletal MM at admission independently predicted falls and mortality 3 months postdischarge in hospitalized older patients [30]. However, in a systematic review the clinical significance of MM was still unknown, due to the small number of longitudinal studies on MM as a predictor of different outcomes [31]. Although the results concerning MM are conflicting, it may be a useful predictor of HRQoL in frail older people whose MM is already very low.

The strengths of our study include its relatively large sample of long-term residents. In addition, our participants were people living their last years, and very few detailed data are available on these subgroups of long-term residents. All the measurements were performed by trained nurses or nutritionists and all the questionnaires and measurements used were validated. Moreover, demographic information, diagnoses, and use of medications and nutritional supplements were retrieved from verified medical records, which increase the reliability of our results. The 15-D questionnaire on HRQoL we used can be completed by proxy and can thus also be used for residents with severe cognitive impairment [18]. The study was not without its limitations, however. Low number of male residents could have affected interpretation of the gender aspects of the study. Body composition was measured with a bioelectrical impedance analysis (BIA) device, because we were unable to use the even more sophisticated dual-energy X-ray absorptiometry (DXA) device, which would have allowed more reliable and detailed results. Finally, the cross-sectional design of the study prevented us from drawing conclusions about temporal relationships.

## Conclusion

Almost all of the residents in our study were at least prefrail, while the majority were identified as frail. MM was strongly correlated with HRQoL in both genders and could therefore be a target for treatment, especially for prefrail participants. Adequate nutritional care combined with physical activity interventions is also encouraged in long-term care to prevent further muscle loss and development of mobility limitations. These interventions, aimed at strengthening and retaining muscle mass of long-term residents, may also improve their HRQoL.
